# Proposals for the Establishment of a Dispensary in This Town, for the Purposes of Supplying the Indigent Sick with Advice and Medicines Gratis, and to Extend the Benefits of Vaccine Inoculation

**Published:** 1806-06

**Authors:** W. Hamilton

**Affiliations:** Halesworth


### *To the Editors of the Medical and Physical Journal.*

GENTLEMEN,

ALTHOUGH the advantages of medical charities have usually been confined in large towns, there seems no reason why they should not be felt, in every place sufficiently populous to support the proper medical attendants; as the expence will necessarily be proportioned to the number of applications.

The following proposals, for the establishment of a Dispensary, have been submitted to the inhabitants of this town and neigbourhood,* and have experienced a very liberal patronage. Their publication may excite others to follow an example, by which, it is hoped, some of the evils of humanity may be alleviated, which induces me to request a place for them in your extensively circulated Journal.

I am, &c.

*Halesworth, May* 9, 1806. W. HAMILTON.

---

*Halesworth.*

*Proposals for the Establishment of a Dispensary in this Town, for the Purposes of supplying the indigent Sick with Advice and Medicines gratis, and to extend the Benefits of Vaccine Inoculation.*

THERE are few, it is presumed, who have made the condition of the poor an object of their attention, in whom it will excite surprise to state, that notwithstanding the relief afforded them by a wise and provident legislature, there are many instances in which it is entirely inadequate, or at best falls far short of the proposed end. Such examples chiefly occur under the pressure of disease, and in the following description of persons:

1. Such as are attacked by disorders requiring speedy aid, at a distance from the parishes upon which they have claims for relief. Such diseases, in very many instances, would readily yield to the early use of appropriate remedies; confirmed by delay, and aggravated by poverty, they often become incurable, and fatal.

2. Those who, enjoying the advantages of speedy assistance,

---

* Halesworth contains about 2,000 inhabitants; the neighbourhood is very populous.

sistance, labour under severe and long-protracted disease, where an extent and variety of expensive remedies may be required; and where, the usual means of relief having failed, a second opinion may be deemed worth resorting to. And,

3. Persons not receiving parochial assistance, but who are nevertheless unable to bear, for any long period, the expences of sickness, superadded to inability to labour, without producing the greatest distress.

Of all the various means by which the charitable and humane have endeavoured to alleviate the distresses arising from these causes, the establishment of dispensaries, where advice and medicines are gratuitously administered, has best attained the proposed end; and the very general adoption of such institutions over every part of the united kingdom, affords the best and most decisive proof of their great utility. It may indeed be safely asserted, that any calculation of the benefit likely to arise from the expenditure of the small sum necessary to defray the annual charge of a well-regulated Dispensary, would fall infinitely below the advantages afforded to the objects of such Institutions.

With these views, the propriety of forming a charity of this nature is respectfully submitted to the inhabitants of this town and neighbourhood, under the following Regulations.

(Dr. Hamilton and Mr. Revans, jun. having offered their services as physician, and surgeon and apothecary to this Institution.)

1. That attendance shall be given by the said physician, and surgeon and apothecary, to such persons as shall apply for relief, upon two days of every week, to be hereafter determined.

2. That this Charity shall be open to all descriptions of persons duly recommended

3. That every yearly subscriber of half a guinea shall have the privilege of recommending any number of patients.

4. That the surgeons and apothecaries resident in the neighbourhood shall be allowed the privilege of recommending patients, although they do not subscribe.

5. That all persons applying for that purpose shall be inoculated for the vaccine disease; aud that a certificate, signed by the physician and surgeon, shall be given to every person that has been duly vaccinated.

To obtain a subscription equal to the demands of an Institution stitution of this kind, will not, it is supposed, be difficult. It is therefore suggested, that the subscriptions should be small; but in order that its benefits may be as widely extended as possible, it is earnestly hoped that the subscribers may be numerous.

When a sufficient number of benevolent persons shall have signified their intentions to subscribe to this institution, there will be a meeting held, in order to agree upon such further rules as may be deemed proper, and to carry the undertaking into immediate effect.

